# Supplementary material for: Diversity of Self-Assembled RNA Complexes: From Nanoarchitecture to Nanomachines
Source: Molecules. 2023 Dec 19;29(1):10. doi: 10.3390/molecules29010010 (PMC10779776; doi:10.3390/molecules29010010)
Supplement: Supplementary file 1 [file molecules-29-00010-s001.zip › molecules-2740157-supplementary.pdf]

Supplementary Materials

Diversity of Self-Assembled RNA Complexes: From Nanoarchitecture to Nanomachines

Maria A. Kanarskaya, Dmitrii V. Pyshnyi and Alexander A. Lomzov *

Institute of Chemical Biology and Fundamental Medicine SB RAS, Novosibirsk 630090, Russia; makanarskaya@gmail.com (M.A.K.); pyshnyi@niboch.nsc.ru (D.V.P.)

*Correspondence: lomzov@niboch.nsc.ru; Tel.: +7-9130092889

HPLC analysis of RNA hydrolysis 1

UV-melting analysis 2

Gel shift assay analysis of the complex types 4

Gel shift assay analysis of nanomachines 9

Molecular dynamics simulation, and analysis 13

HPLC analysis of RNA hydrolysis

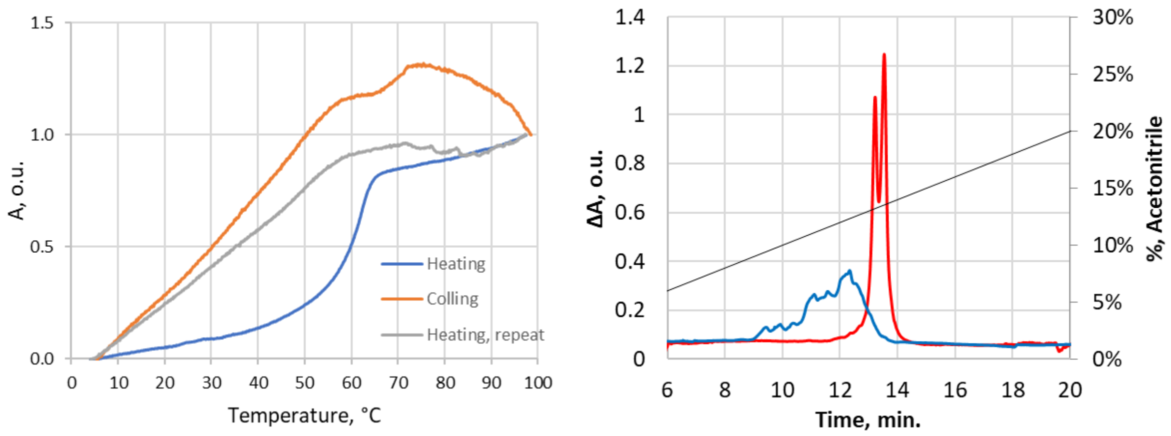

Figure S1. UV melting curves of M/N-U3 complex during heating, cooling and heating (left). RP-HPLC analysis of the complex before (red line) and after (blue line) the UV-melting experiment (right).

UV-melting analysis

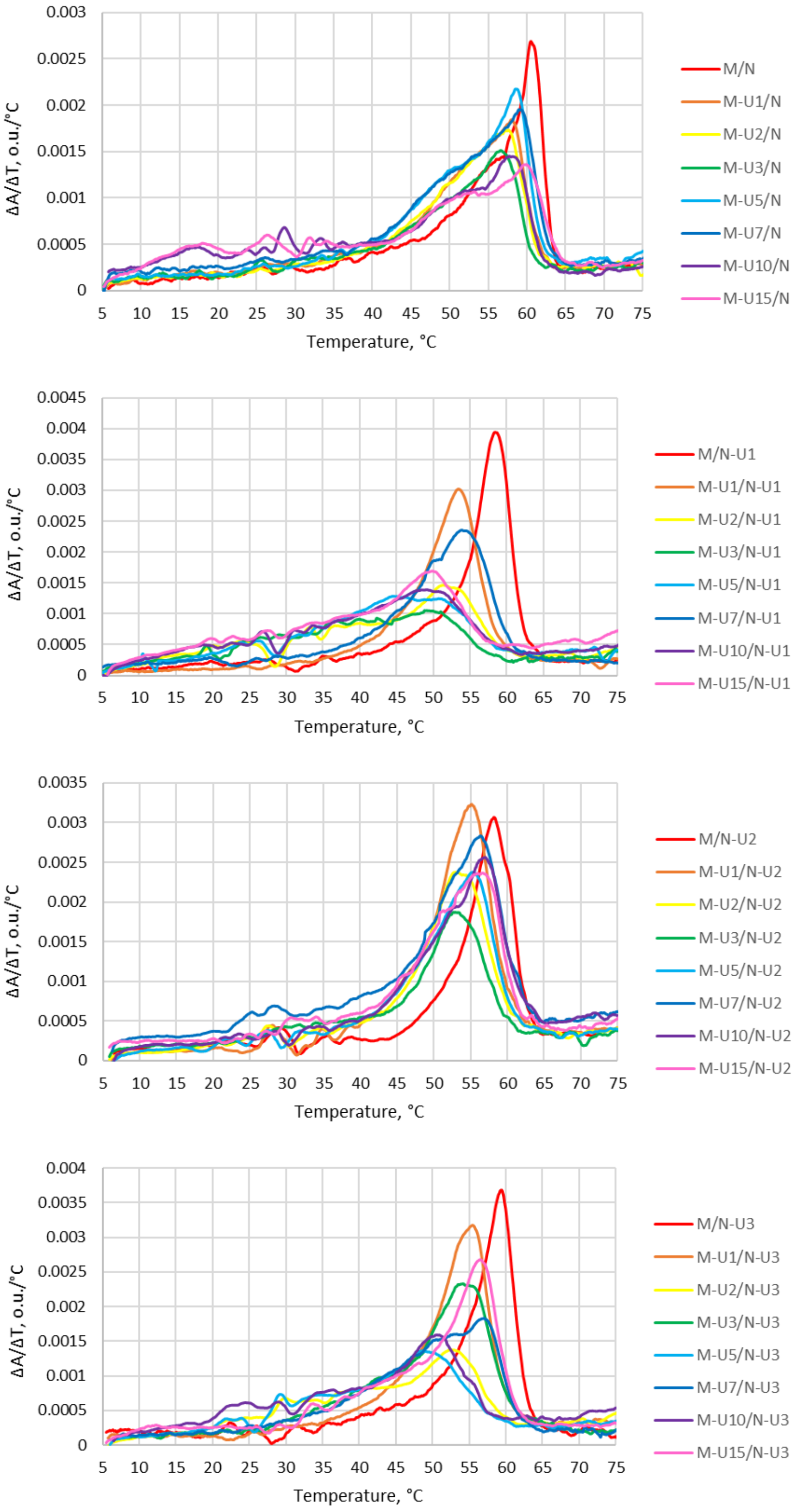

Figure S2. Differential UV melting curves of M-Ui/N-Uj complexes (i = 0, 1, 2, 3, 5, 7, 10, and 15; j = 0, 1, 2, and 3).

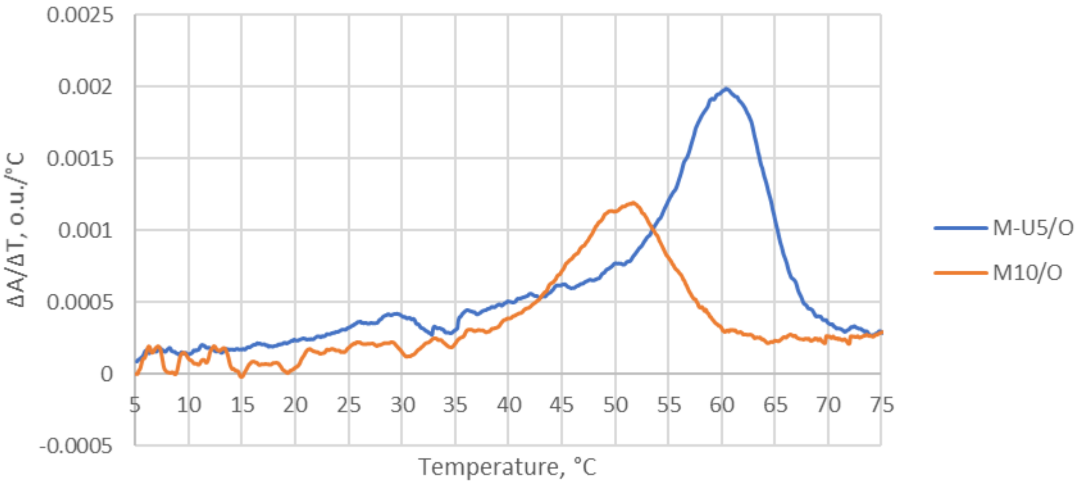

Figure S3. Differential UV melting curves of M-U5/O and M10/O complexes.

Table S1. Melting temperatures (Tm) of the complexes are determined as a maximum of differential UV melting curves.

Complex	Tm, °C	Complex	Tm, °C
M/O	54.0	M10/O	51.8
M-U1/O	57.0	N-U3/DNA[1]	44.0
M-U2/O	59.0	O/C	59.0
M-U3/O	59.0	M-U5/OL	69.0
M-U5/O	60.8	OL/CL	73.0

[1] DNA opener 5′-d(CCATCATATGAAAAA)-3′

Gel shift assay analysis of the complex types

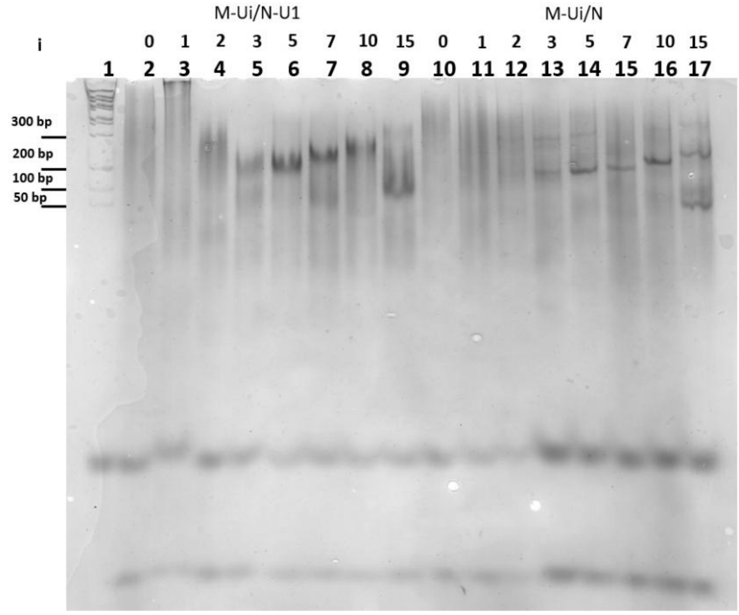

Table S2. Mobility, complex type, and size determined by analysis gel shift assays.

Lane	Complex	Mobility, bp	Complex type/size
1	Ladder	-	
2	M/N-U1	-	Conc
3	M-U1/N-U1	-	Conc
4	M-U2/N-U1	c,280	Conc,4
5	M-U3/N-U1	(100), 220	(2),4
6	M-U5/N-U1	220	4
7	M-U7/N-U1	(100),250	(2),4
8	M-U10/N-U1	280	4
9	M-U15/N-U1	100, (300)	2(4)
10	M/N	-	Conc
11	M-U1/N	-	Conc
12	M-U2/N	c,(200), (300)	Conc, (4), (6)
13	M-U3/N	c,200, 300	Conc, 4, 6
14	M-U5/N	c,200, 300	Conc, 4, 6
15	M-U7/N	c,200	Conc, 4
16	M-U10/N	c,220, (300)	Conc, 4, (6)
17	M-U15/N	50, 220, (350)	2, 4, (6)

Figure S4. The gel shift assay of oligonucleotides' complexes M-Uj/N-U1 and M-Uj/N, j = 0, 1, 2, 3, 5, 7, 10, and 15. The order of complexes in electropherogramm, complexes' name, complex mobility, and type are shown in the Table S2. A dsDNA ladder of 50–1000 bp is shown on the left.

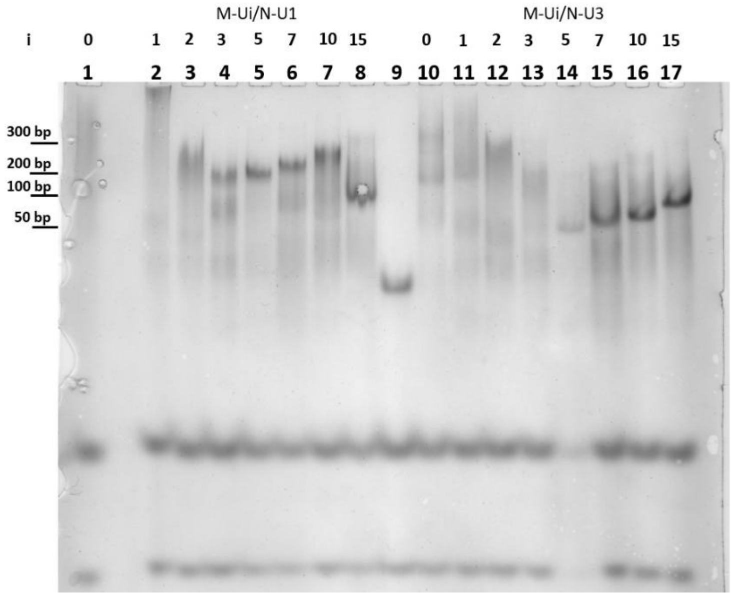

Table S3. Mobility, complex type, and size determined by analysis gel shift assays.

Lane	Complex	Mobility, bp	Complex type/size
1	M/N-U1	C	Conc
2	M-U1/N-U1	C	Conc
3	M-U2/N-U1	c,280	Conc,4
4	M-U3/N-U1	(100), 220	(2),4
5	M-U5/N-U1	220	4
6	M-U7/N-U1	(100),250	(2),4
7	M-U10/N-U1	280	4
8	M-U15/N-U1	100,(300)	2,(4)
9	M10/N10	10	duplex
10	M/N-U3	c, 50, 200, 350	Conc, 2, 4, 6
11	M-U1/N-U3	c, 50, 200	Conc, 2, 4
12	M-U2/N-U3	c,40, 300	2,6
13	M-U3/N-U3	220,(300)	4,(6)
14	M-U5/N-U3	40	2
15	M-U7/N-U3	50	2
16	M-U10/N-U3	50	2
17	M-U15/N-U3	90	2

Figure S5. The gel shift assay of oligonucleotides' complexes M-Uj/N-U1 and M-Uj/N-U3, j = 0, 1, 2, 3, 5, 7, 10, and 15. The order of complexes in electropherogramm, complexes' name, complex mobility, and type are shown in the Table S3. A dsDNA ladder of 50–1000 bp is shown on the left.

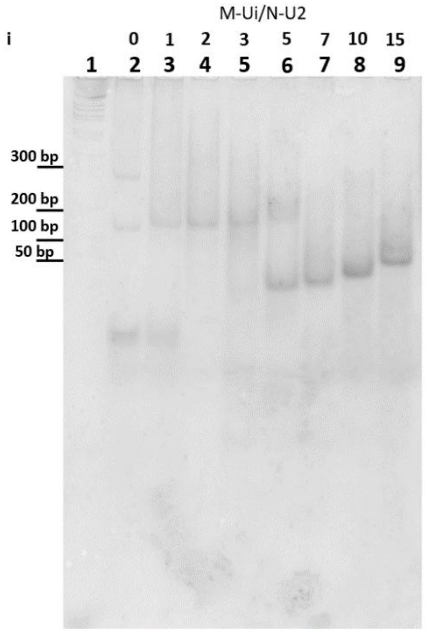

Table S4. Mobility, complex type, and size determined by analysis gel shift assays

Lane	Complex	Mobility, bp	Complex type/size
1	Маркер	-	
2	M/N-U2	180, 280	Conc, 4, 6
3	M-U1/N-U2	180	Conc, 4
4	M-U2/N-U2	180	Conc, 4
5	M-U3/N-U2	(50), 180	Conc, 4
6	M-U5/N-U2	30, (180)	2, (4)
7	M-U7/N-U2	30	2
8	M-U10/N-U2	40	2
9	M-U15/N-U2	50	2

Figure S6. The gel shift assay of oligonucleotides' complexes M-Uj/N-U2, j = 0, 1, 2, 3, 5, 7, 10, and 15. The order of complexes in electropherogramm, complexes' name, complex mobility, and type are shown in the Table S4. A dsDNA ladder of 50–1000 bp is shown on the left.

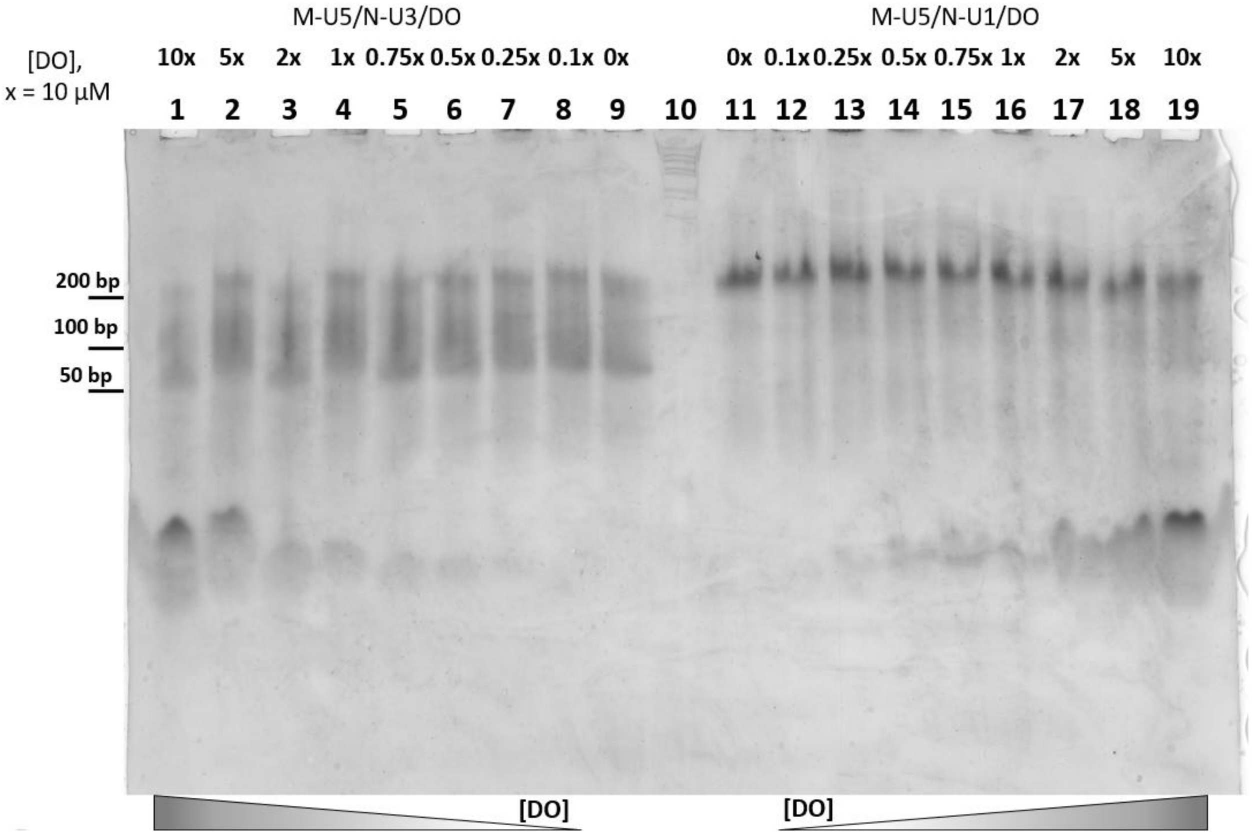

Figure S7. Confirmation of self-limited of complex formation. Gel shift assays of RNA complexes (M-U5/N-U1 and M-U5/N-U3) in the presence of DNA opener 5′-d(CCATCATATGAAAAA)-3′ DO at different concentrations. Lanes: 1, M-U5/N-U3/DO (1 : 1 : 10); 2, M-U5/N-U3/DO (1 : 1 : 5); 3, M-U5/N-U3/DO (1 : 1 : 2); 4, M-U5/N-U3/DO (1 : 1 : 1); 5, M-U5/N-U3/DO (1 : 1 : 0.75); 6, M-U5/N-U3/DO (1 : 1 : 0.5); 7, M-U5/N-U3/DO (1 : 1 : 0.25); 8, M-U5/N-U3/DO (1 : 1 : 0.1); 9, M-U5/N-U3 (1 : 1); 10. lader; 11, M-U5/N-U1 (1 : 1); 12, M-U5/N-U1/DO (1 : 1 : 0.1); 13, M-U5/N-U1/DO (1 : 1 : 0.25); 14, M-U5/N-U1/DO (1 : 1 : 0.5); 15, M-U5/N-U1/DO (1 : 1 : 0.75); 16, M-U5/N-U1/DO (1 : 1 : 1); 17, M-U5/N-U1/DO (1 : 1 : 2); 18, M-U5/N-U1/DO (1 : 1 : 5); 19, M-U5/N-U1/DO (1 : 1 : 10). In the brackets the ration of components concentration in the sample noted. Value 1 corresponded to 10 μM. A dsDNA ladder of 50–1000 bp is shown on the left.

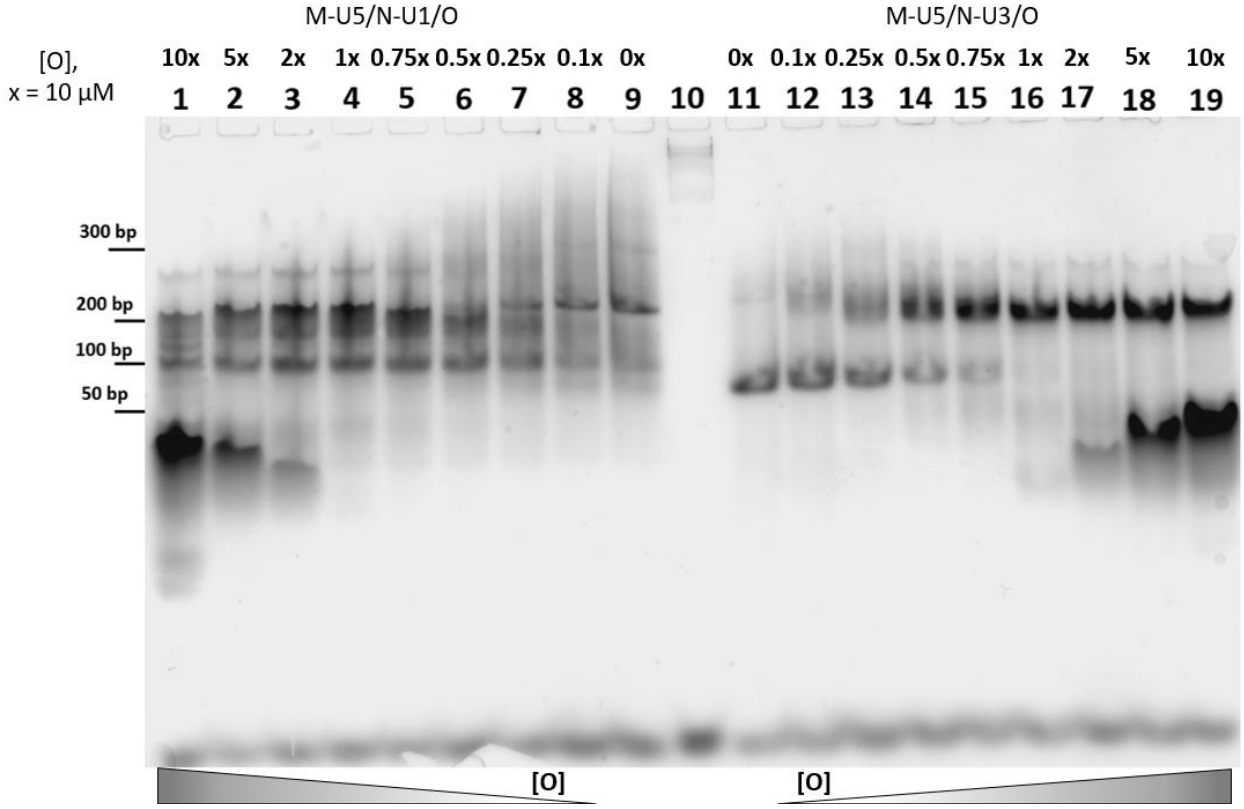

Figure S8. Determination of self-limited complex molecularity. Gel shift assays RNA complexes (M-U5/N-U1 and M-U5/N-U3) in the presence of RNA-opener O. Lanes: 1, M-U5/N-U1/O (1 : 1 : 10); 2, M-U5/N-U1/O (1 : 1 : 5); 3, M-U5/N-U1/O (1 : 1 : 2); 4, M-U5/N-U1/O (1 : 1 : 1); 5, M-U5/N-U1/O (1 : 1 : 0.75); 6, M-U5/N-U1/O (1 : 1 : 0.5); 7, M-U5/N-U1/O (1 : 1 : 0.25); 8, M-U5/N-U1/O (1 : 1 : 0.1); 9, M-U5/N-U1 (1 : 1); 10, ladder; 11, M-U5/N-U3 (1 : 1); 12, M-U5/N-U3/O (1 : 1 : 0.1); 13, M-U5/N-U3/O (1 : 1 : 0.25); 14, M-U5/N-U3/O (1 : 1 : 0.5); 15, M-U5/N-U3/O (1 : 1 : 0.75); 16, M-U5/N-U3/O (1 : 1 : 1); 17, M-U5/N-U3/O (1 : 1 : 2); 18, M-U5/N-U3/O (1 : 1 : 5); 19, M-U5/N-U3/O (1 : 1 : 10). In the brackets the ration of components concentration in the sample noted. Value 1 corresponded to 10 µM. A dsDNA ladder of 50–1000 bp is shown on the left.

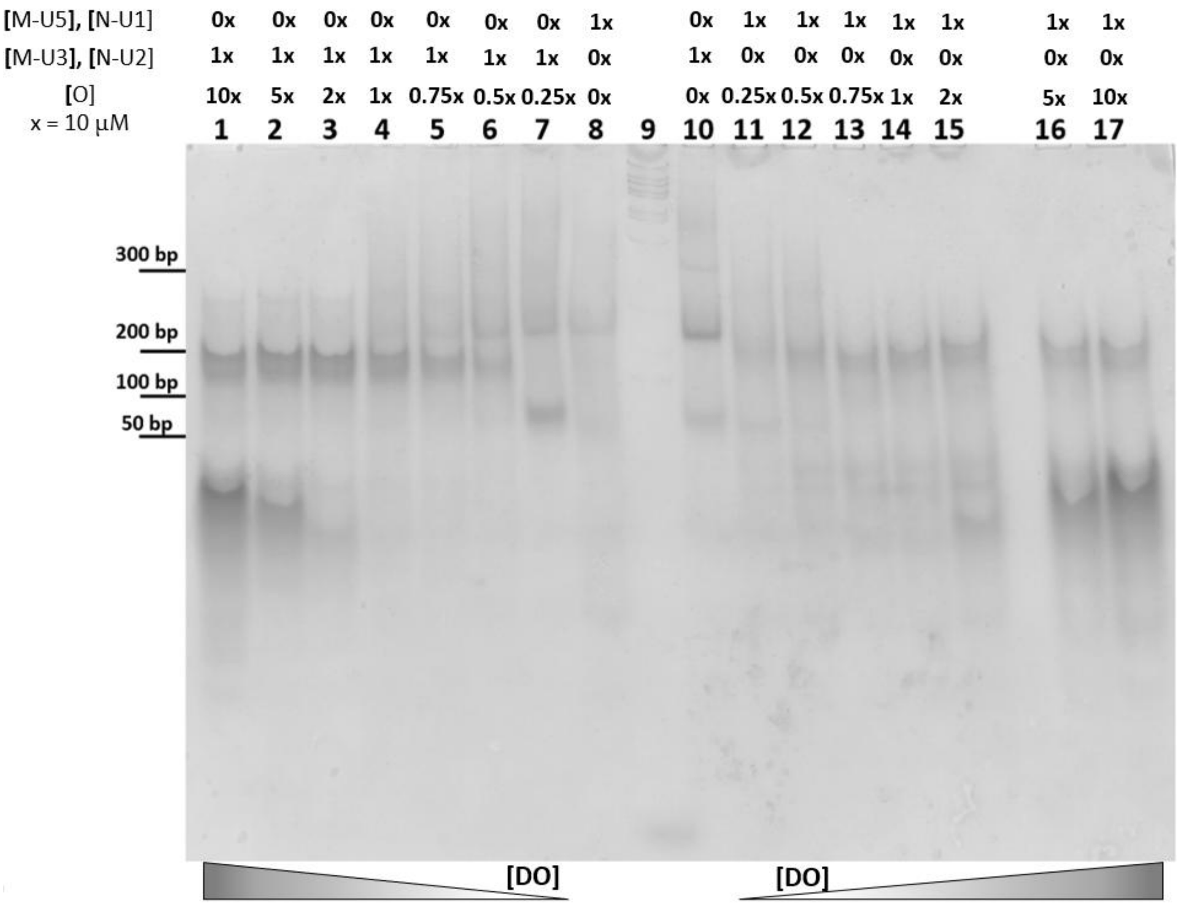

Figure S9. Determination of self-limited complex molecularity. Gel shift assays RNA complexes (M-U5/N-U1 and M-U3/N-U2) in the presence of RNA-opener O. Lanes: 1, M-U5/N-U1/O (1 : 1 : 10); 2, M-U5/N-U1/O (1 : 1 : 5); 3, M-U5/N-U1/O (1 : 1 : 2); 4, M-U5/N-U1/O (1 : 1 : 1); 5, M-U5/N-U1/O (1 : 1 : 0.75); 6, M-U5/N-U1/O (1 : 1 : 0.5); 7, M-U5/N-U1/O (1 : 1 : 0.25); 8, M-U3/N-U2 (1 : 1); 9, ladder; 10, M-U5/N-U1 (1 : 1); 11, M-U3/N-U2/O (1 : 1 : 0.25); 12, M-U3/N-U2/O (1 : 1 : 0.5); 13, M-U3/N-U2/O (1 : 1 : 0.75); 14, M-U3/N-U2/O (1 : 1 : 1); 15, M-U3/N-U2/O (1 : 1 : 2); 16, M-U3/N-U2/O (1 : 1 : 5); 17, M-U3/N-U2/O (1 : 1 : 10). In the brackets the ration of components concentration in the sample noted. Value 1 corresponded to 10 μM. A dsDNA ladder of 50–1000 bp is shown on the left.

Gel shift assay analysis of nanomachines

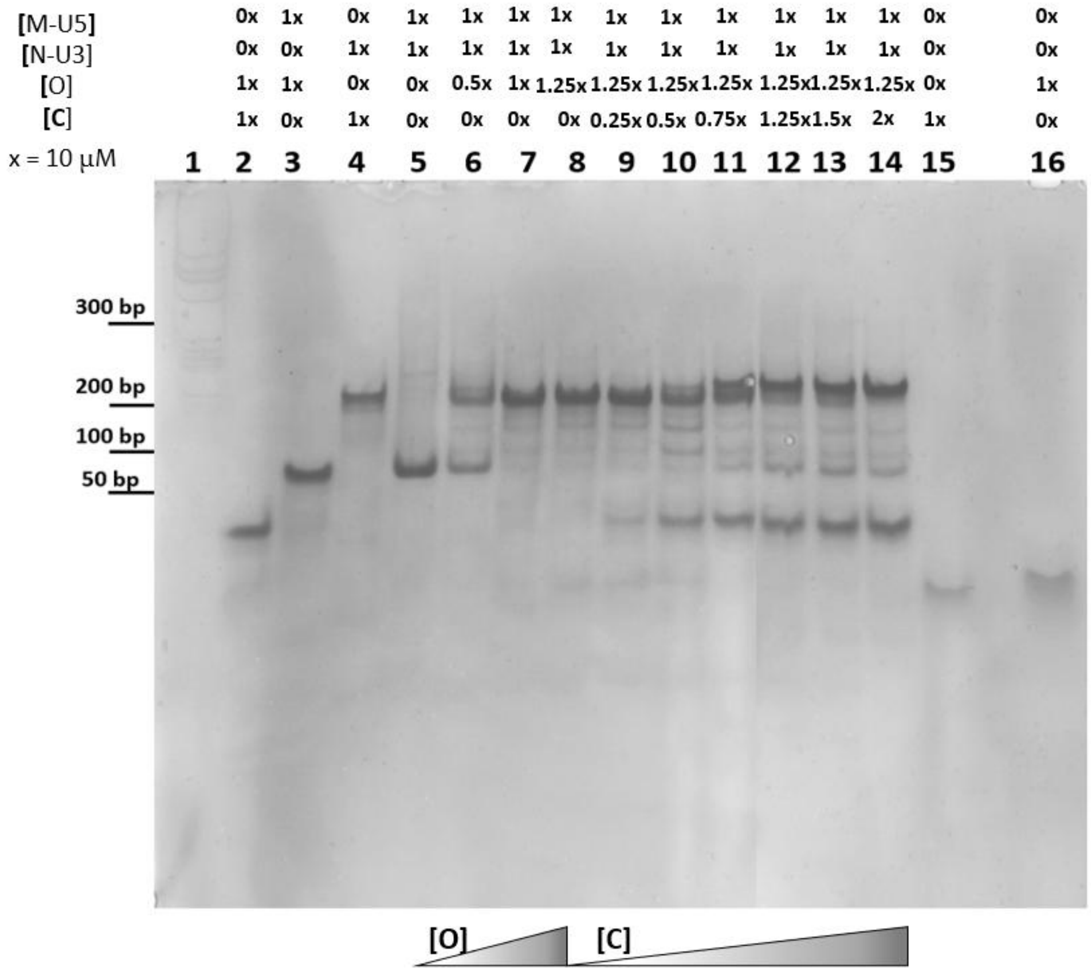

Figure S10. Determination of the possibility of M-U5/N-U3 complex “opening” and “closing” by adding O and C oligonucleotides, respectively. Gel shift assays of RNA complexes M-U5/N-U3 in the presence of RNA opener O and closer C at different concentrations. Lanes: 1, ladder; 2, C/O (1:1); 3, M-U5/O (1:1); 4, C/N-U3 (1:1); 5, M-U5/N-U3 (1 : 1); 6, M-U5/N-U3/O (1 : 1 : 0.5); 7, M-U5/N-U3/O (1 : 1 : 1); 8, M-U5/N-U3/O (1 : 1 : 1.25); 9, M-U5/N-U3/O/C (1 : 1 : 1,25 : 0.25); 10, M-U5/N-U3/O/C (1 : 1 : 1.25 : 0.5); 11, M-U5/N-U3/O/C (1 : 1 : 1.25 : 0.75); 12, M-U5/N-U3/O/C (1 : 1 : 1.25 : 1.25); 13, M-U5/N-U3/O/C (1 : 1 : 1.25 : 1.5); 14, M-U5/N-U3/O/C (1 : 1 : 1.25 : 2); 15, C (1); 16, O (1). The concentration of every component is equal to 10 μM marked as 1. A dsDNA ladder of 50–1000 bp is shown on the left.

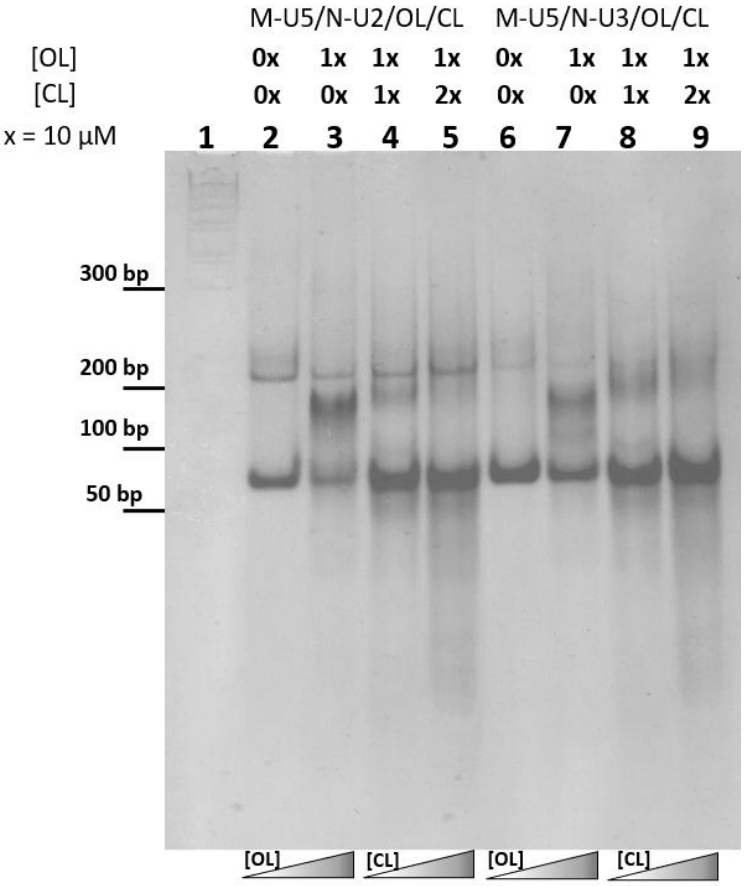

Figure S11. Determination of the possibility of M-U5/N-U2 and M-U5/N-U3 complex "opening" and "closing" by adding elongated OL and CL oligonucleotides, respectively. Gel shift assays of RNA complexes M-U5/N-U3 in the presence of RNA opener OL and closer CL at different concentrations. Lanes: 1, ladder; 2, M-U5/N-U2 (1 : 1); 3, M-U5/N-U2/OL (1 : 1 : 1); 4, M-U5/N-U2/OL/CL (1 : 1 : 1 : 1); 5, M-U5/N-U2/OL/CL (1 : 1 : 1 : 2); 6, M-U5/N-U3 (1 : 1); 7, M-U5/N-U3/OL (1 : 1 : 1); 8, M-U5/N-U3/OL/CL (1 : 1 : 1 : 1); 9, M-U5/N-U3/OL/CL (1 : 1 : 1 : 2). The concentration of every component is equal to 10 μM marked as 1. A dsDNA ladder of 50–1000 bp is shown on the left.

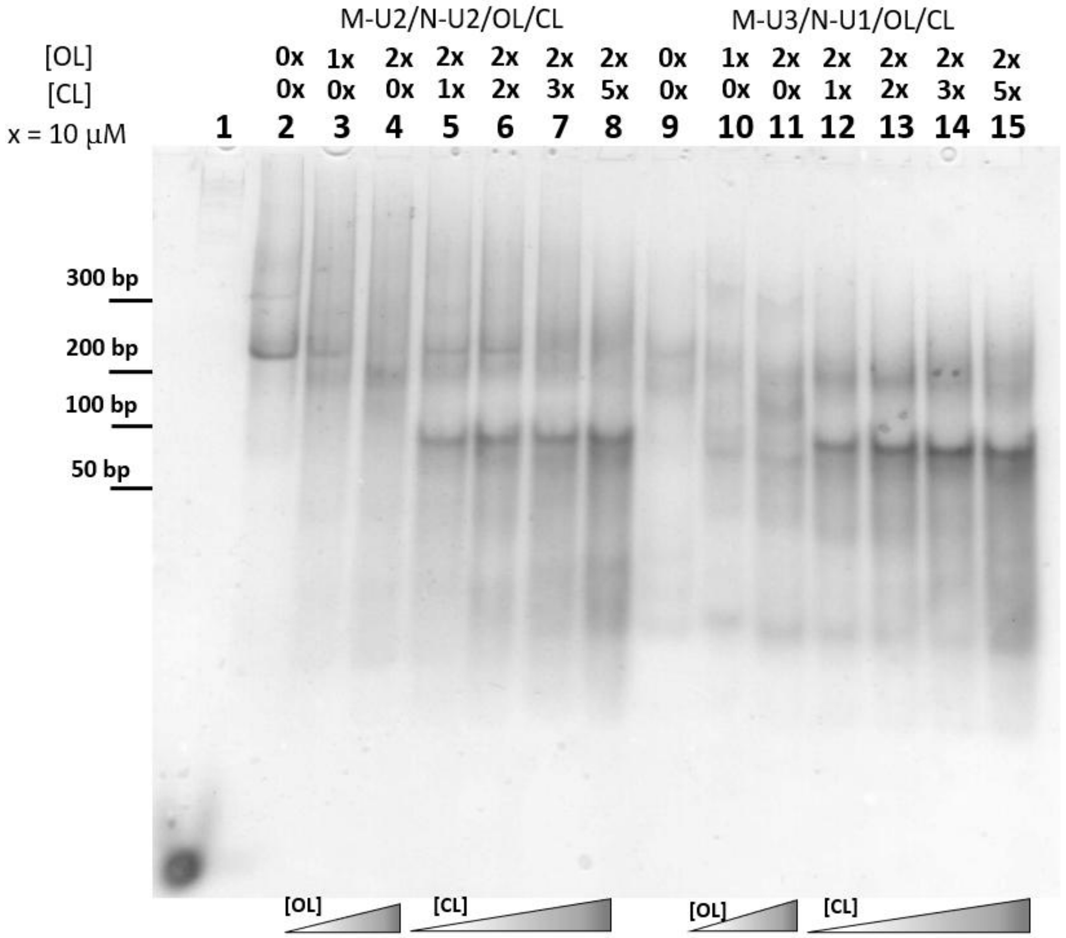

Figure S12. Determination of the possibility of M-U2/N-U2 and M-U3/N-U1 complex "opening" and "closing" by adding elongated OL and CL oligonucleotides, respectively. Gel shift assays of RNA complexes in the presence of RNA opener OL and closer CL at different concentrations. Lanes: 1, ladder; 2, M-U2/N-U2 (1 : 1); 3, M-U2/N-U2/OL (1 : 1 : 1); 4, M-U2/N-U2/OL (1 : 1 : 2); 5, M-U2/N-U2/OL/CL (1 : 1 : 2 : 1); 6, M-U2/N-U2/OL/CL (1 : 1 : 2 : 2); 7, M-U2/N-U2/OL/CL (1 : 1 : 2 : 3); 8, M-U2/N-U2/OL/CL (1 : 1 : 2 : 5); 9, M-U3/N-U1 (1 : 1); 10, M-U3/N-U1/OL (1 : 1 : 1); 11, M-U3/N-U1/OL (1 : 1 : 2); 12, M-U3/N-U1/OL/CL (1 : 1 : 2 : 1); 13, M-U3/N-U1/OL/CL (1 : 1 : 2 : 2); 14, M-U3/N-U1/OL/CL (1 : 1 : 2 : 3); 15, M-U3/N-U1/OL/CL (1 : 1 : 2 : 5). The concentration of every component equal to 10 μM marked as 1. A dsDNA ladder of 50–1000 bp is shown on the left.

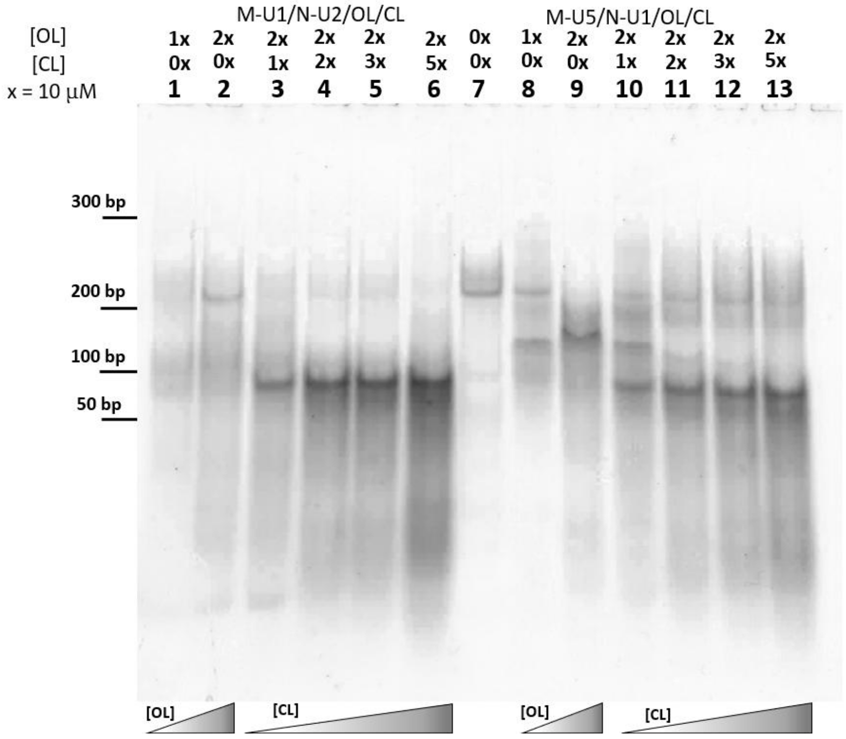

Figure S13. Determination of the possibility of M-U1/N-U2 and M-U5/N-U1 complex "opening" and "closing" by adding elongated OL and CL oligonucleotides, respectively. Gel shift assays of RNA complexes in the presence of RNA opener OL and closer CL at different concentrations. Lanes: 1, M-U1/N-U2/OL (1 : 1 : 1); 2, M-U1/N-U2/OL (1 : 1 : 2); 3, M-U1/N-U2/OL/CL (1 : 1 : 2 : 1); 4, M-U1/N-U2/OL/CL (1 : 1 : 2 : 2); 5, M-U1/N-U2/OL/CL (1 : 1 : 2 : 3); 6, M-U1/N-U2/OL/CL (1 : 1 : 2 : 5); 7, M-U5/N-U1 (1 : 1); 8, M-U5/N-U1/OL (1 : 1 : 1); 9, M-U5/N-U1/OL (1 : 1 : 2); 10, M-U5/N-U1/OL/CL (1 : 1 : 2 : 1); 11, M-U5/N-U1/OL/CL (1 : 1 : 2 : 2); 12, M-U5/N-U1/OL/CL (1 : 1 : 2 : 3); 13, M-U5/N-U1/OL/CL (1 : 1 : 2 : 5). The concentration of every component equal to 10 μM marked as 1. A dsDNA ladder of 50–1000 bp is shown on the left.

Molecular dynamics simulation, and analysis

M-U3/N-U2

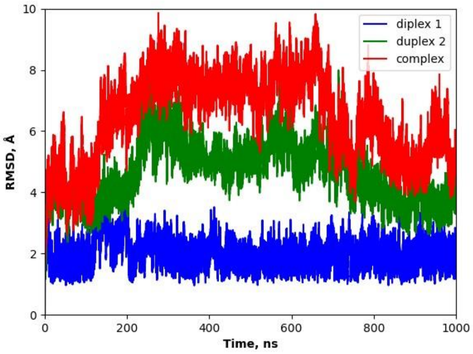

M-U5/N-U2

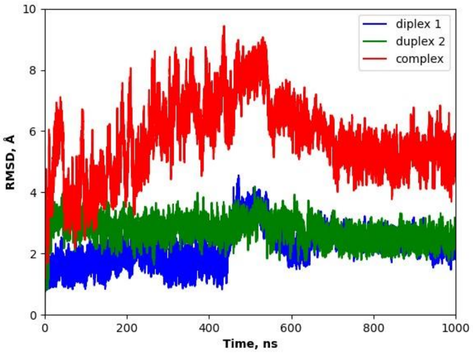

$(M\text{-}U3/N\text{-}U2)_2$

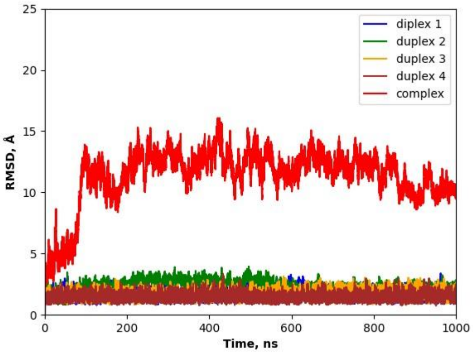

$(M\text{-}U3/N\text{-}U3)_2$

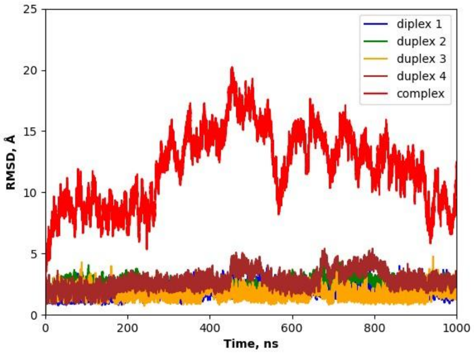

$(M\text{-}U3/N\text{-}U2)_3$

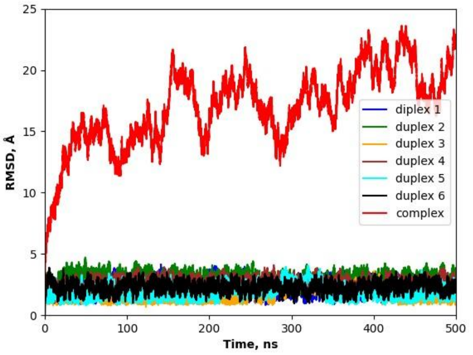

Figure S14. RMSD values along the MD trajectories for the complexes studied.

a b

c d

e

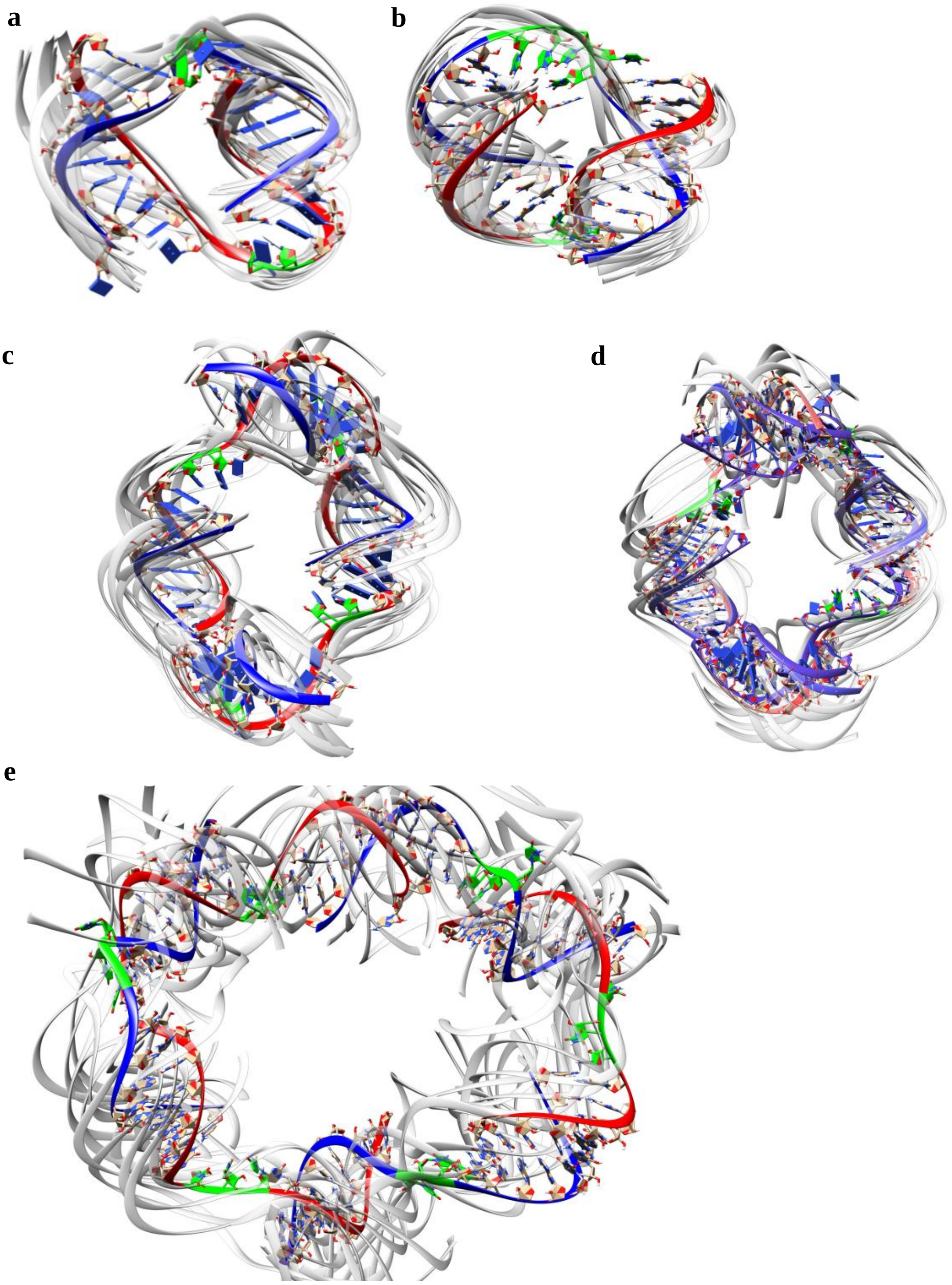

Figure S15. Superimposing of ten most represented in the trajectory strictures RMSD of complexes obtained by hierarchical cluster analysis: values along the MD trajectories for the complexes studied: (a) M-U3/N-U2, (b) M-U5/N-U2, (c) $(M\text{-}U3/N\text{-}U2)_2$, (d) $(M\text{-}U3/N\text{-}U3)_2$, (e) $(M\text{-}U3/N\text{-}U3)_6$. Most representative structures in the trajectories are shown in colors: linkers are shown as green, oligonucleotides of the M series are shown with blue backbone, and N series with red backbone. All other structures are shown as gray ribbons.
